# First Insights on Early Host Plants and Dispersal Behavior of *Halyomorpha halys* (Hemiptera: Pentatomidae) from Overwintering to Crop Colonization

**DOI:** 10.3390/insects11120866

**Published:** 2020-12-06

**Authors:** Lara Bosco, Martina Nardelli, Luciana Tavella

**Affiliations:** Dipartimento di Scienze Agrarie, Forestali e Alimentari (DISAFA), University of Torino, Largo P. Braccini 2, I-10095 Grugliasco, TO, Italy; lara.bosco@unito.it (L.B.); nardellimartina94@gmail.com (M.N.)

**Keywords:** brown marmorated stink bug, hazelnut crop, pheromone-baited trap, immunomarking-capture technique, north-western Italy

## Abstract

**Simple Summary:**

Following its first detection in North Italy in 2012, *H. halys* has become a serious threat in many crops, including hazelnut. Since favorite host plants and dispersal behavior of overwintered adults of *H. halys* before crop colonization are little known, research was carried out in four polyculture areas (from 14 to 50 ha) in north-western Italy in 2018, by using (i) pheromone-baited traps, (ii) visual inspection and beating sheet sampling, and (iii) immunomarking-capture technique. After overwintering, adults responded to pheromone; the host plants of the same species hosted higher numbers of *H. halys* when they were near a pheromone trap. Adults were capable of both short- and long-range dispersal from overwintering sites and/or early host plants to wild and crop plants. Their movement patterns depended on the ecosystem features, and plant host distribution and availability. This knowledge together with the interaction between pheromone and early host plants might contribute to the management of post-overwintering adults before crop colonization.

**Abstract:**

Following its first detection in North Italy in 2012, *H. halys* has become a serious threat in many crops, including hazelnut. The present study aimed at investigating dispersal capacity and behavior in relation to host plants of overwintered adults of *H. halys* before the colonization of hazelnut crop. Research was carried out in four polyculture areas (from 14 to 50 ha) in north-western Italy in 2018, by using (i) pheromone-baited traps, (ii) visual inspection and beating sheet sampling, and (iii) immunomarking-capture technique. The relative abundance of *H. halys* was similar between and within the study areas, and the early attractiveness of lures to adults after overwintering was confirmed; the host plants near a pheromone trap (less than 5 m) hosted higher numbers of *H. halys* than the same plant species far away. Hybrid plane, European spindletree, walnut, oak, and European elder were the first plants on which adult bugs were observed to feed. By immunomarking-capture technique, *H. halys* showed both short- and long-range dispersal from overwintering sites and/or early host plants to wild and crop plants. Marked adults were found in all zones of each area, irrespective of the distance from the protein treatment. Therefore, *H. halys* movement patterns depend on the ecosystem features, and plant host distribution and availability. This knowledge together with the interaction between pheromone and early host plants might contribute to the management of post-overwintering adults.

## 1. Introduction

The brown marmorated stink bug *Halyomorpha halys* (Stål) (Hemiptera: Pentatomidae) is an invasive pest that in the last few decades has expanded its range, colonizing new areas, and now poses a serious threat to many agricultural crops [1]. Following its accidental introduction to North America and Europe, *H. halys* has continued its expansion to many other areas, such as Turkey and Chile [2]. After its first detection in North Italy in 2012, *H. halys* has become the main pest in many crops, especially apple, nectarine, pear, and hazelnut [3,4], causing serious economic losses and nullifying the IPM strategies widely adopted in the area, due to the high numbers of treatments, usually with broad spectrum insecticides [5], required to manage this pest.

Like most phytophagous pentatomids, *H. halys* is polyphagous with a broad host range that includes over 170 plants in a wide range of plant families, many of agricultural importance, including various fruit, vegetables, row crops, and ornamentals [1,6,7]. Although *H. halys* feeds and often disperses on a wide range of plants during the season, hosts vary in suitability and acceptability. Both adults and nymphs prefer to feed on plant reproductive structures, which are often required to complete development [1]. In addition, multiple host plant species are needed for optimal *H. halys* development [8]. *Halyomorpha halys* also has a strong dispersal capacity; adults can fly long distances and spread at landscape level to locate mature fruit to feed on [9,10], while both adults and nymphs are also capable of walking dispersal [11].

During the growing season, *H. halys* frequently moves between crop and wild plants following temporal patterns of fruit ripening. For this reason, it often invades fields from wild hosts, and is usually more abundant along crop field edges [4,12,13,14]. Moreover, this insect often disperses between different habitats in pursuit of its preferred hosts, and like many hemipterans, starts its dispersal from overwintering sites to host plants in early spring [6]. *Halyomorpha halys* adults overwinter in concealed, sheltered locations, including beneath the bark of dead upright trees, in rocky outcroppings, and in a variety of human-made structures, especially those located in rural landscapes [5,7,15,16].

In north-western Italy, adults usually start to exit from overwintering sites in March–early April, with numbers peaking in late spring (May–early June) depending on environmental conditions. Little is known about their behavior at this time; host plant resources are limited, whereas their flight capacity is high, therefore resulting in long-distance dispersal of *H. halys* [9]. Where adults go immediately after leaving overwintering sites is little explored; although, in Asia, they are reported to utilize arboreal hosts [17]. Given the difficult management of this pest once it colonizes a crop [1], a better understanding of its preferred host plants and of its dispersal behavior in early spring may assist in developing successful pest management strategies. Since alternative host plants are significant for stink bug development and can be essential for population build-up before dispersing to agricultural crops [18], identification of early and alternative host plants can be exploited to develop farm scale programmes aimed at *H. halys* control.

In addition to visual inspection and beating sheet sampling, pheromone-baited traps can be used to monitor infestation levels and dispersal of *H. halys*. The male-produced *H. halys* aggregation pheromone is a mixture of two stereoisomers of 10,11-epoxy-1-bisabolen-3-ol (aggregation pheromone, hereafter). Traps have been baited with this aggregation pheromone and methyl (E,E,Z)-2,4,6-decatrienoate (MDT, hereafter), which resulted in an increased effect on field attraction of both adults and nymphs [19]. In fact, this combination has captured all life stages and both sexes of *H. halys* in the USA, Europe, and Asia throughout the growing season [19,20]. For a better understanding of the dispersal capacity and behavior of *H. halys*, mark-recapture studies using immunomarking technique have also been employed [12,21]. Marking insects in situ with a field-applied protein marker minimally impacts dispersal over space and time. Insects are "marked" with a unique protein, either by direct contact during application or by picking up the protein via contact with previously marked surfaces, and subsequently analyzed for the specific protein by enzyme-linked immunosorbent assay (ELISA) [22,23,24].

The present study aimed, therefore, at investigating the dispersal capacity and behavior of overwintered adults of *H. halys* in relation to host plants before the colonization of hazelnut crop, which is one of the most important crops in north-western Italy, as well as one of the most damaged by *H. halys* [4]. Specifically, research was carried out in four polyculture areas in north-western Italy, by using (i) pheromone-baited traps to monitor *H. halys* populations leaving the overwintering refuges for crop colonization, (ii) visual inspection and beating sheet sampling to assess its distribution and abundance on early host plants, and (iii) an immunomarking-capture technique to track its movement and dispersal behavior from overwintering refuges and/or early host plants before hazelnut crop colonization at the landscape scale.

## 2. Materials and Methods

### 2.1. Sites and Study Design

Trials were performed in four polyculture areas located in Piedmont, north-western Italy, in 2018. Each area was 14 to 50 ha and included different crops and natural habitats, both uncultivated corridors and forest margins (Table 1). In each area, relatively high infestation levels of *H. halys* on crops were recorded during the previous season; moreover, high numbers of overwintering bugs were also recovered in the closest human structures (buildings, woodsheds, storehouses, and heaps of rubble). The study design was conceived for each area in order to include one hypothetical overwintering site, usually a human building, approximately in the middle of the area.

Six pheromone traps were arranged radially around the hypothetical overwintering site, with a radius of 150–200 m in Area 1 (14 ha), 300–400 m in Area 2 (28 ha), and 400–500 m in Areas 3 and 4 (48 and 50 ha, respectively). Distance from the area centre and traps varied taking into account the size and shape of cultivated plots, in order to place the traps on the border of crop plots, and not in their centre. SAGA GIS-Module Grid Proximity program was used to calculate the Euclidian distance between each trap and the closest feature considered: (i) a source of water (i.e., river); (ii) main road suitable for vehicles (i.e., secondary suburban road); and (iii) building (Table 1). The differences in the captures between the six traps in relation to these features were investigated.

To assess early host plants and track movement of *H. halys*, in each area, from 8 to 9 zones were selected because of one or more of the following characteristics: (i) presence of host plants for stink bug sampling, either in natural habitats or cultivated plots; (ii) presence of the pheromone trap; (iii) presence of potential overwintering refuges, either human-made or natural, and/or early host plants to be used for the immunomarking study. Zones were tagged with capital letters (A–H or A–I) and are reported for each area in Figure S1. 

### 2.2. Sampling by Pheromone Traps

In each area, the pheromone traps were placed starting from early/mid-April (Table 1). Traps consisted of a transparent sticky card (153 × 305 mm), horizontally set on a cane or a tree branch, baited with a lure consisting of 20 mg of aggregation pheromone and 200 mg of MDT (Trécé, Inc., Adair, OK, USA). Following the manufacturer’s instructions, during the sampling period, the lure dispensers were changed once after 12 weeks, while the sticky cards were replaced several times, depending on the number of trapped insects and on weather conditions. From April until June or early July, depending on the area (Table 1), traps were checked from 6 to 8 times, at variable intervals (between 7 and 14 days). During each sampling, stink bug adults and, when present, nymphs were removed as described below, and transferred to the laboratory.

The mean numbers of daily captures of males, females and nymphs by pheromone traps were calculated. They were compared firstly within each area for sampling date and for zone; then they were compared between areas, across the sampling period. One-way ANOVA was performed, after tests of homogeneity of variance (Levene) and normality (Shapiro–Wilk), and means were then separated at *p* < 0.05 using Tukey’s test (IBM SPSS Statistics 25).

### 2.3. Sampling on Host Plants

During surveys between early/mid-April and mid-/late June, wild and cultivated host plants in each area were identified and inspected for the presence of *H. halys*. Stink bugs were sampled by visual inspection, followed by scouting branches or stems with a cane on a beating sheet (0.7 × 0.7 m). Due to the different size and shape, the plants were visually inspected for 1 min and then scouted on sheet five times (sampling unit). Moreover, data of sampling were attributed to 10-day intervals for each month as follows: (I) from 1st to 10th day; (II) from 11th to 20th day; and (III) from 21st to 30th or 31st day.

Mean numbers of stink bug adults collected per plant (sampling unit) in the four areas were analyzed for differences in relation to (i) the fruiting or vegetative stage of plants, both for the whole period and for each single period; (ii) the lure proximity (< 5 m) across the whole period; (iii) the major crops from May to late June. After tests of homogeneity of variance (Levene) and normality (Shapiro–Wilk), one-way ANOVA was performed for points (i) and (ii), whereas the non-parametric Kruskal–Wallis test was used for point (iii) because data were inhomogeneous (IBM SPSS Statistics 25).

### 2.4. Immunomarking-Capture Technique

To track the movement of bugs between overwintering sites, early host plants and crops, an immunomarking-capture technique was adopted. The two unique protein-markers, albumin and casein, were used in the form of tap water solution of 10% liquid egg white and 20% skimmed milk, respectively. The protein solutions were misted on host plants or overwintering sites, with a 14 L motorized backpack sprayer (SR450, Stihl S.p.A., Cambiago, Italy). In particular, milk solution was used to mark possible anthropic overwintering sites such as shelters, piles of rubbles and woodshed, while egg white solution was misted on possible natural overwintering sites or early host plants, such as oak, ivy, and black locust. Only in Area 2, protein solutions were used to mark wild hazelnut in the forest side (albumin) and cultivated hazelnut in the orchard (casein) (Table 2).

During surveys, adults were individually collected from traps using an individual disposable plastic stick and from plants by hand, wearing disposable gloves. Each adult was then sexed, and placed into an individual 5 mL centrifuge tube, labelled with date, area, zone, trap or plant, and sex.

In the laboratory, indirect enzyme-linked immunoadsorbent assays (ELISA) were performed to detect the presence of markers (casein and albumin) on the samples. During each assay, adults were simultaneously analyzed for the presence of each protein, by using two 96-well microplates (Thermo Fisher Scientific, Waltham, MA, USA), one for casein and one for albumin. At the same time, *H. halys* adults collected from traps in a site 10 km away from the closest experimental area were used both directly as negative controls, and after soaking them in a solution of milk or egg white with plastic disposable tweezers, as positive controls. Each microplate was loaded with 29 samples of adults (3 wells for each sample), and the relative positive, negative, and buffer-only controls, each repeated in three wells.

Analyses were performed following a slightly modified protocol described by Blaauw et al. [10], Jones et al. [20] and Lessio et al. [21]. Rabbit anti-casein (RAC, GTX37769, GeneTex, Irvine, CA, USA; 16 μL into 8 mL of PBSS-BS20 for each microplate) and rabbit anti-egg (RAE, C6534, Sigma-Aldrich, St. Louis, MO, USA; 2 μL into 8 mL PBSS-BS20 for each microplate) were used as the primary antibody for casein and albumin, respectively. For both egg white and milk assays, donkey anti-rabbit (DAR, A120-108P, Bethyl Laboratories, Inc., Montgomery, TX, USA; 1.4 μL into 8.4 mL PBSS-BS20 for each microplate) was used as the secondary antibody. PBST (Phosphate buffered saline + 0.09% Triton X-100, Triton-X-100, Sigma-Aldrich, St. Louis, MO, USA) and PBS-SDS (Phosphate buffered saline + 2.3 g L^−1^ sodium dodecyl sulfate) were used as washing solutions. TBS-EDTA (Tris-buffered saline pH 8.0 + 0.3 g L^−1^ ethylenediaminetetraacetic acid, Sigma-Aldrich, St. Louis, MO, USA), PBSS-BS20 (Phosphate buffered saline + 20% bovine serum + 1300 ppm Silweet L-77, Silwet, Chemtura Manufacturing, Manchester, UK) and 2N H_2_SO_4_ were used as reagents. Ultra-TMB (34028, Thermo Fisher Scientific, Rockford, IL, USA) was used as substrate solution. 

Immunomarking-capture results of each area were evaluated by considering the distances between the sampling zones and the zone of treatment. Sampling dates were pooled together, when the captured adults were considered to have undergone the same treatment or a repetition of it (approximately in the one-month interval). Adults collected in the same area, zone and sampling period were grouped. To compare data among the four areas, each sampling date was attributed to a 10-day interval as explained above. Percentages of samples marked with albumin, or with casein, or with both, or unmarked were arcsine square-root-transformed and analyzed with one-way ANOVA, using areas, zones and 10-day periods as the main factor. Means were separated at *p* < 0.05 using Tukey’s test (IBM SPSS Statistics 25).

## 3. Results

### 3.1. Population Dynamics after Overwintering Assessed by Pheromone Traps

**Figure 1:**
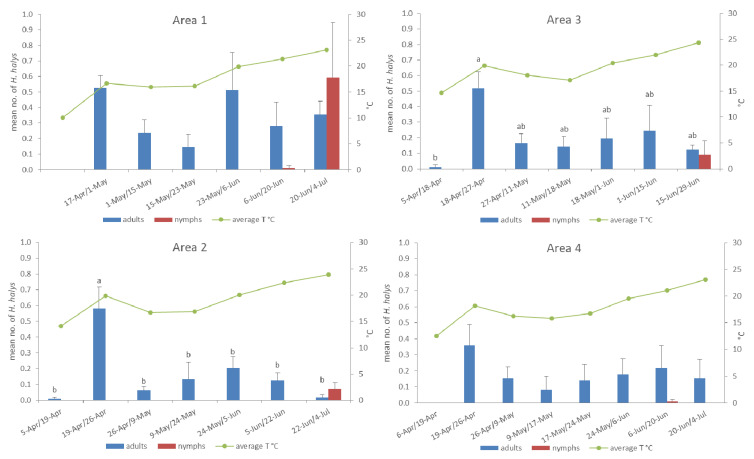
Mean numbers of daily captures of adults and nymphs of *Halyomorpha halys* per trap and average temperatures (°C) recorded in each area in the sampling period. Bars of adult captures labelled with the same letter are not significantly different (one-way ANOVA, Tukey’s test, *p* < 0.05).

Cumulatively, 392 adults and 51 nymphs were captured with pheromone traps in the four areas from April to late June–early July (Table 1). The mean number of daily captures for each area is reported in Figure 1. Overall, mean daily captures ranged from 0.01 to 0.58, with a peak occurring in late April, concurrently with an unusual increase in the average temperature (18.6 °C). Nymphs appeared in all areas in June. Significant differences in mean daily captures of adults between sampling dates were recorded by one-way ANOVA in Area 2 (F (6, 35) = 7.168, *p* < 0.0001) and Area 3 (F (6, 35) = 2.672, *p* = 0.031). No significant differences were found in Area 1 (F (5, 30) = 1.371, *p* = 0.263) and Area 4 (F (7, 40) = 1.044, *p* = 0.417) (Figure 1).

Among the six traps the range of distances fluctuated: (i) from a minimum of 117 m in Area 4 to a maximum of 556 m in Area 2 for the closest source of water; (ii) from 131 m in Area 1 to 754 m in Area 4 for the closest main roads; and (iii) from 150 m in Area 1 to 251 m in Area 4 for the closest buildings (Table 1). Results of one-way ANOVA on mean daily captures by traps in relation to the distances from the closest source of water, main roads and buildings did not show any significant difference, in the numbers of males, females, adults (males + females), nymphs and total population (adults + nymphs) (*p* > 0.05, data not shown). Therefore, the considered features did not seem to explain any variation in *H. halys* numbers.

The mean number of daily captures by traps was used to compare all the four areas. Despite higher captures (0.38 adults + nymphs/trap/day) in Area 1, there were no significant differences among the four areas by one-way ANOVA during the period from April to late June–early July (adults + nymphs: F (3, 20) = 2.199, *p* = 0.120).

### 3.2. Early Colonization and Abundance on Host Plants

Floral composition, including crops (Table 1) and wild plants, was different in the four study areas. In particular, some host plants were present in almost every area, whereas others were linked to a specific area, like bay tree (*Laurus nobilis* L.), hybrid plane (*Platanus x hispanica* Mill ex Münchh.), false indigo-bush (*Amorpha fruticosa* L.), fig (*Ficus carica* L.), peach (*Prunus persica* (L.) Batsch) and American pokeweed (*Phytolacca americana* L.) (Table 3). In total, 491 *H. halys* bugs (462 adults and 29 nymphs) were sampled on plants in the four areas between mid-April and mid-/late June. As few nymphs were collected in the sampling period, only adults were considered in the following analyses; moreover, adults sampled on plants located nearby (less than 5 m) pheromone lures were not considered here. Qualitative evaluation on the numbers of adults per plant collected across the sampling period revealed that peach, common ash (*Fraxinus excelsior* L.), red mulberry (*Morus rubra* L.), apple (*Malus domestica* Borkh.), maple (*Acer* spp.) hybrid plane and hazelnut (*Corylus avellana* L.) were major host plants (from 15.3 to 2.8 adults per plant collected in total), followed by cherry (*Prunus avium* (L.) L.), ivy (*Hedera helix* L.), walnut (*Juglans regia* L.), poplar (*Populus* spp.), and European spindletree (*Euonymus europaeus* L.). Hybrid plane, European spindletree, oak (*Quercus* spp.), walnut, and European elder (*Sambucus nigra* L.) were the first host plants of the season, on which adults were collected in April. In contrast, *H. halys* was never collected on hawthorn (*Crataegus monogyna* Jacq.) (Areas 1, 2, 4), rose (*Rosa canina* L.) (Areas 1, 2), lime (*Tilia* spp.) (Area 1), Asian knotweed (*Reynoutria japonica* Houtt.) (Area 3), privet (*Ligustrum* sp.) (Area 3), and sunflower (*Helianthus annuus* L.) (Area 3). Both adults and nymphs were frequently found on maize (*Zea mays* L.) but only later in the season.

**Figure 2:**
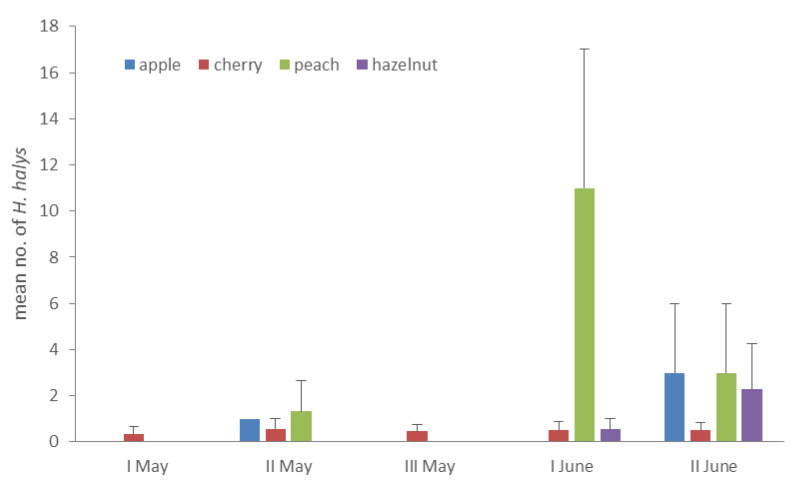
Mean numbers (±SE) of *Halyomorpha halys* adults per plant on the crops sampled from early May to mid-June.

Host plants were evaluated for the presence of fruit during the sampling period. In total, 14 species were bearing fruit at different ripening stages on at least one sampling date, whereas the remaining species were present in a vegetative stage (Table 3). Independently of the period, the presence of fruit resulted a significant factor for the mean density of *H. halys* (mean numbers of adults per plant per sampling date) (ANOVA: F (1, 22) = 12.358, *p* = 0.002). Differences in abundance of *H. halys* between fruiting or vegetative stages were recorded in the following 10-day periods: II May (ANOVA: F (1, 24) = 6.722, *p* = 0.016), I June (ANOVA: F (1, 21) = 5.371, *p* = 0.031), and II June (ANOVA: F (1, 21) = 17.581, *p* < 0.0001). Among fruiting plant species, crops presenting fruit from early May to late June, i.e., apple, cherry, peach, and hazelnut, were compared for *H. halys* abundance. Although on cherry seasonal abundance was quite stable across the sampling period, on other crops population levels generally fluctuated (Figure 2). Nevertheless, no significant differences were found between these four crops in the mean values pooled across the sampling period I May–II June (Kruskal–Wallis: df = 3; χ^2^ = 3.053; *p* = 0.383; *N* = 22).

**Figure 3:**
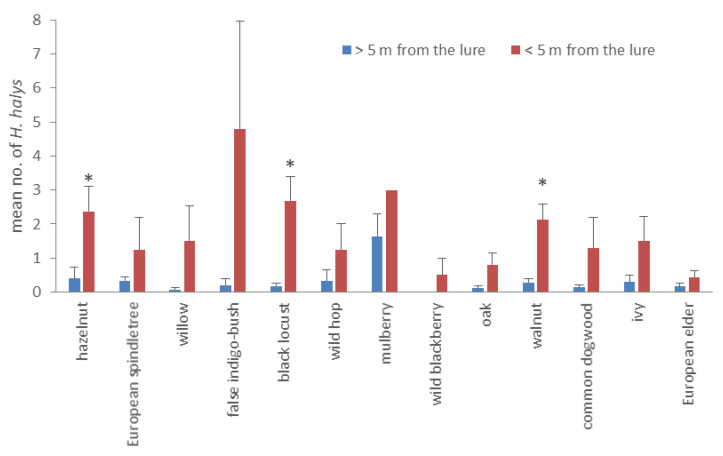
Mean numbers (±SE) of *Halyomorpha halys* adults per plant, sampled on same species located either in the proximity (5 m) of the pheromone lure or farther (> 5 m) from it. * indicates that significant differences in mean number of *H. halys* were recorded for that plant species (one-way ANOVA, *p* < 0.05).

Some host plant species (no. 13) were located also in the proximity (i.e., ≤ 5 m) of the lures (Figure 3). Due to the potential interaction between the lure and the host plant, these plant species were considered separately from the same species farther away. Overall, mean values of the adults collected across the sampling period were significantly higher on all the plants located within 5 m from the lures compared to all the plants of the same species located farther from the lures (ANOVA: F (1, 28) = 18.051, *p* < 0.0001). Although each species nearby hosted higher values of *H. halys* adults compared to the same species far away, significant differences were recorded only on hazelnut (ANOVA: F (1, 11) = 6.670, *p* = 0.025), black locust (*Robinia pseudoacacia* L.) (ANOVA: F (1, 11) = 14.203, *p* = 0.003) and walnut (ANOVA: F (1, 11) = 17.279, *p* = 0.002) (Figure 3). The sampling periods (no. 7) did not affect the factor proximity/distance from the lure (ANOVA; F (6, 145) = 1.659, *p* = 0.135).

Starting from early June, the first nymphs appeared on host plants and were found in low numbers on common ash, walnut, red mulberry, common wheat (*Triticum aestivum* L.), black locust, false indigo-bush, European spindletree, common dogwood (*Cornus sanguinea* L.) and hazelnut (data not shown).

### 3.3. Movement and Crop Colonization Tracked with Immunomarking-Capture

Overall, 597 adults collected either on traps or on host plants in the four areas were analyzed by ELISA. Of these, 30% was albumin positive, 14% was casein positive, and 9% was positive for both markers. The mean percentages of marked and unmarked specimens grouped per sampling date from April to June, and per sampling zone, were not significantly different among the four investigated areas (ANOVA: albumin: F (3, 104) = 1.866, *p* = 0.140; casein: F (3, 110) = 1.942, *p* = 0.127; albumin + casein: F (3, 104) = 1.991, *p* = 0.120; unmarked: F (3, 110) = 2.116, *p* = 0.102), neither between the zones in the investigated areas (ANOVA: albumin: F (7, 100) = 0.462, *p* = 0.860; casein: F (7, 106) = 0.845, *p* = 0.553; albumin + casein: F (7, 100) = 0.294, *p* = 0.955; unmarked: F (7, 106) = 0.653, *p* = 0.711).

**Figure 4:**
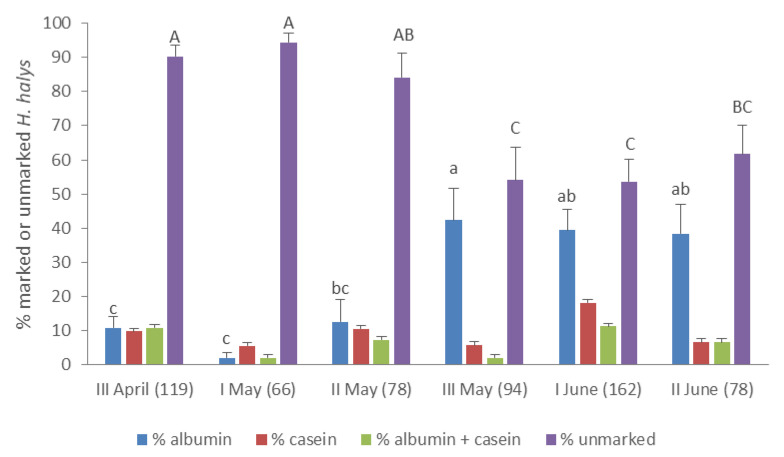
Mean percentages (±SE) of marked and unmarked *Halyomorpha halys* adults captured in the four study areas from late April to mid-June. Lower case and upper case letters indicate significant differences between 10-day periods for albumin positive and unmarked specimens, respectively (Tukey’s test following one-way ANOVA, *p* < 0.05). On the x-axis, for each 10-day periods, the total number of tested specimens is given in brackets.

Across the sampling period, significant differences were found in the percentages of specimens unmarked and marked with albumin (ANOVA: unmarked: F (5, 108) = 7.167, *p* < 0.0001; albumin: F (5, 102) = 6.571, *p* < 0.0001), whereas no significant differences were recorded in the percentages of specimens marked with casein and with both proteins (ANOVA: casein: F (5, 108) = 2.136; *p* = 0.067; albumin + casein: F (5, 102) = 1.952, *p* = 0.092) (Figure 4).

**Figure 5:**
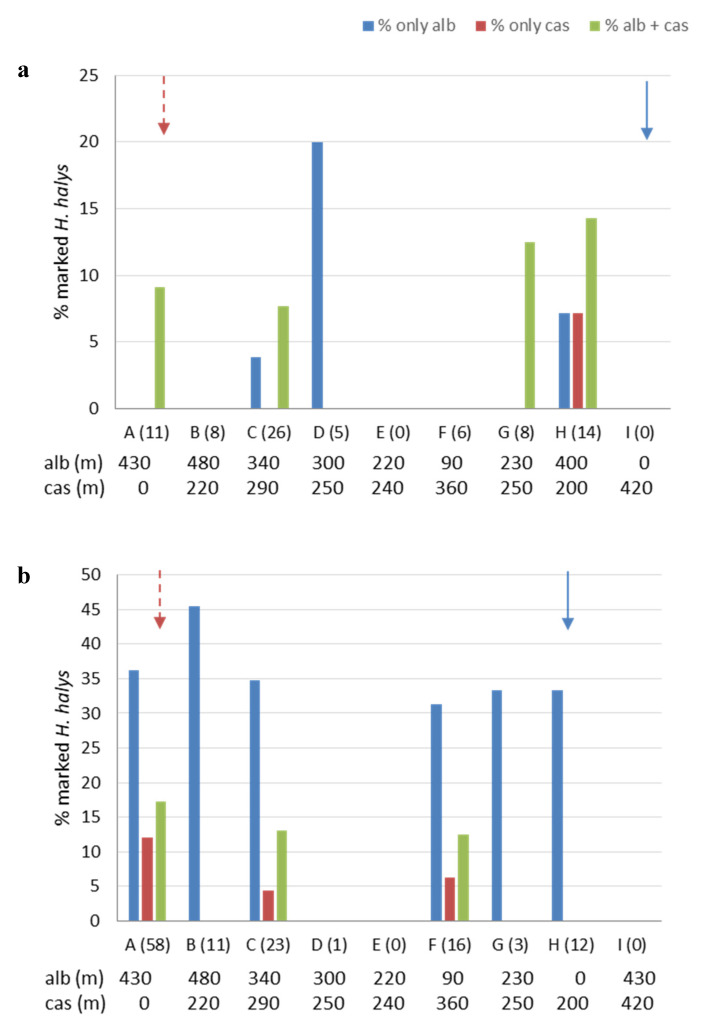
Total percentages of marked *Halyomorpha halys* adults collected in the sampling zones (A–H) of Area 1 from 17 April to 15 May (**a**) and from 16 May to 18 June (**b**). On the x-axes, for each zone, the total number of tested specimens is given in brackets. For each zone, the distance (meters) from the treatment site is also indicated either for albumin (“alb”) or casein (“cas”). Arrows designate the zones in which treatments with casein (dashed dark red arrow) and albumin (blue arrow) were applied.

The percentages of marked *H. halys* collected in the nine zones of Area 1, for the period from 17 April to 15 May and from 16 May to 18 June are reported in Figure 5a,b, respectively. Following the first treatment with milk solution in potential overwintering refuges (Table 2), adults marked with casein were found in zones at distances of 0 to 290 m, in higher numbers in zones H and G (200 and 250 m, respectively). The first treatment with egg white solution was made in the hazelnut orchard (Table 2), and adults marked with albumin were found in zones up to 430 m away, in higher numbers in zones H and D (400 and 300 m, respectively) (Figure 5a). Following the second treatments with milk and egg white solution on peach and oak, respectively (Table 2), adults marked with casein were found only in three zones up to 360 m, with the highest amounts in the zone of treatment, while adults marked with albumin were more numerous (on average 36%) compared to the first period (Figure 5b), and found in zones up to 420 m. The 6% of tested adults were positive to both casein and albumin in both sampling periods.

**Figure 6:**
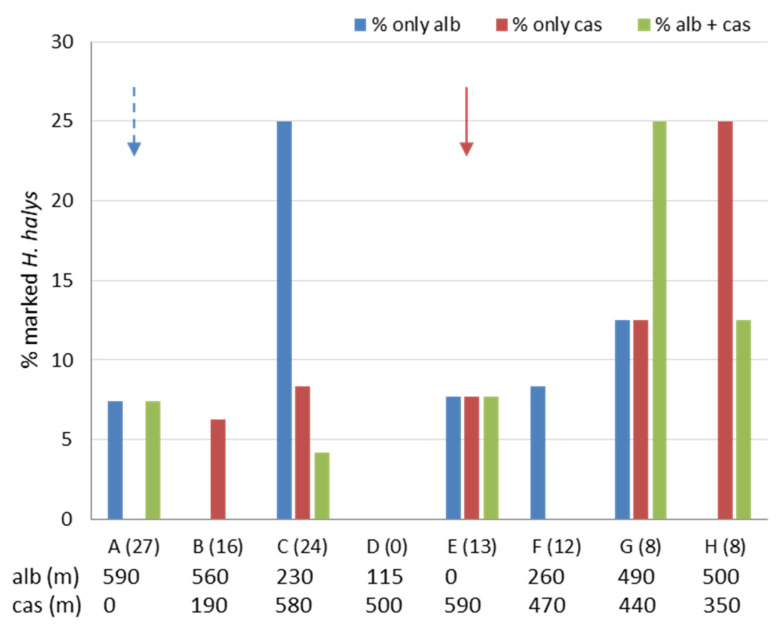
Total percentages of marked *Halyomorpha halys* adults collected in the sampling zones (A–H) of Area 2 from 26 April to 5 June. On the x-axis for each zone the total number of tested specimens is given in brackets. For each zone, the distance (meters) from the treatment site is also indicated either for albumin (“alb”) or casein (“cas”). Arrows designate the zones in which treatments with casein (dashed dark red arrow) and albumin (blue arrow) were applied.

In Area 2, one treatment with each marker was applied, therefore only the period from 26 April to 5 June was considered. Milk solution was sprayed in a hazelnut orchard (Table 2), and adults marked with casein were found in zones up to 590 m, in higher numbers in zones G and H (440 and 350 m, respectively) (Figure 6). Egg white solution was sprayed on wild hazelnut (Table 2), and marked adults were found at distances up to 590 m, in higher numbers in zones G and C (490 and 230 m, respectively) (Figure 6). Total percentages of marked adults were equal for casein (17%) and albumin, whereas on average 8% of tested adults were positive for both casein and albumin.

**Figure 7:**
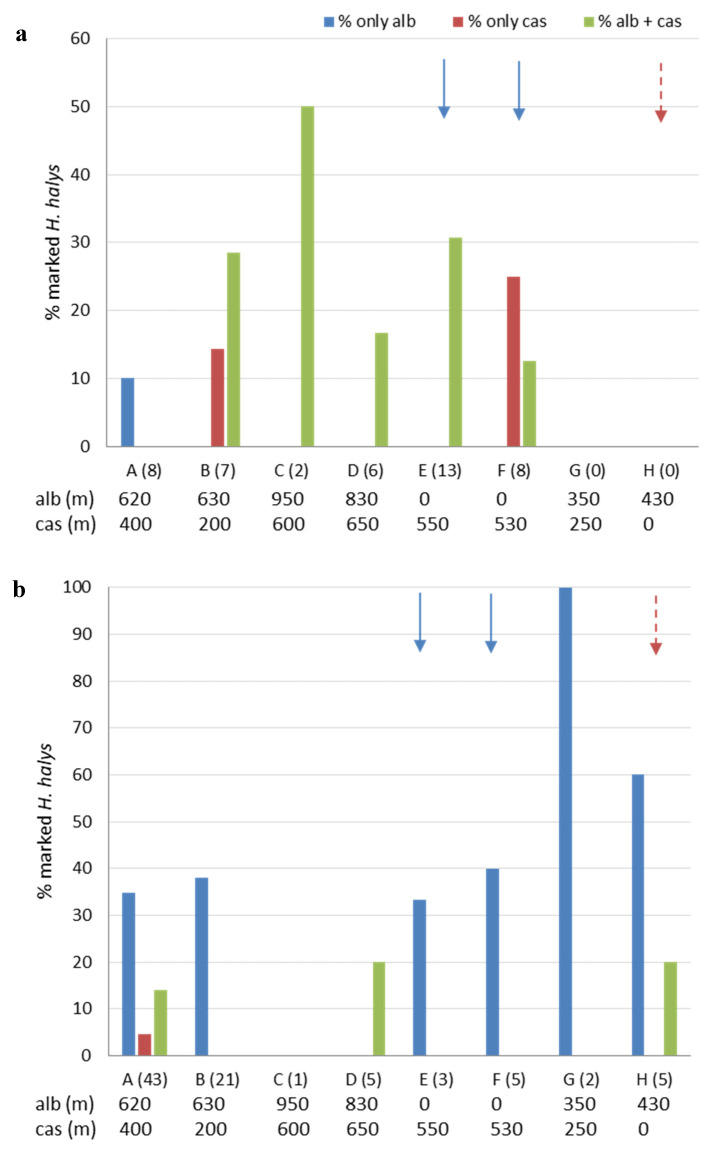
Total percentages of marked *Halyomorpha halys* adults collected in the sampling zones (A–H) of Area 3 from 5 April to 27 April (**a**) and from 28 April to 30 May (**b**). On the x-axes for each zone the total number of tested specimens is given in brackets. For each zone, the distance (meters) from the treatment site is also indicated either for albumin (“alb”) or casein (“cas”). Arrows designate the zones in which treatments with casein (dashed dark red arrow) and albumin (blue arrow) were applied.

The percentages of marked *H. halys* in the eight zones of Area 3, for the period from 5 to 27 April and from 28 April to 30 May are reported in Figure 7a,b, respectively. In April, almost all specimens (43 out of 44) were collected on 27 April, after both treatments with casein in a potential overwintering site and the treatment with albumin on plants considered as early hosts of *H. halys* (Table 2). Adults marked with casein or marked with both albumin and casein were found up to 650 m and 950 m away from the zone of treatment, respectively. Nevertheless, none of the tested adults was positive only to albumin (Figure 7a). In late April–May, adults marked with casein (either casein alone or casein and albumin) were found in three out of the eight zones, but at distances up to 650 m from treatment site (Figure 7b). From the first to the second period, total percentages of adults marked with casein decreased (from 27% to 7%), whereas those with albumin increased (from 23% to 45%). On average 23% and 7% of tested adults were positive to both casein and albumin in the first and second period, respectively. Moreover, adults that were positive to one or both markers were found up to 42 days after the treatment on 18 April.

**Figure 8:**
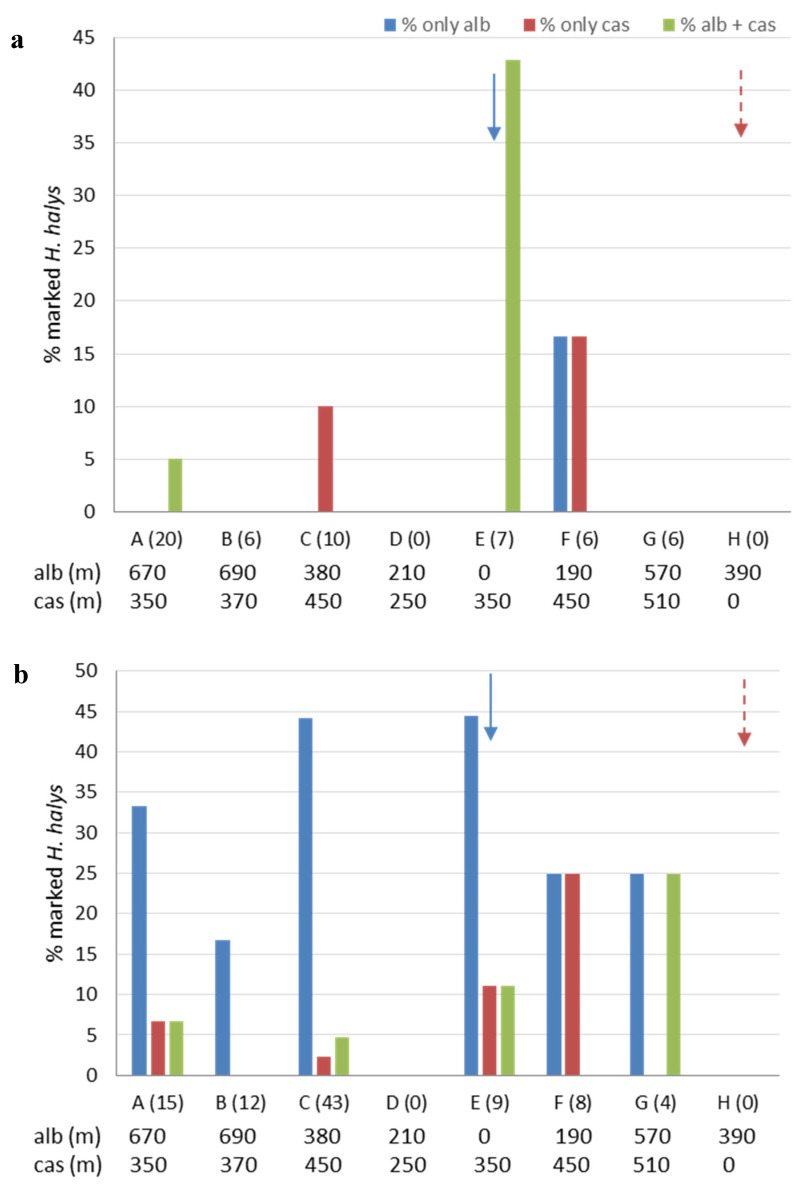
Total percentages of marked *Halyomorpha halys* adults collected in the sampling zones (A–H) of Area 4 from 19 April to 17 May (**a**) and from 18 May to 22 June (**b**). On the x-axes for each zone the total number of tested specimens is given in brackets. For each zone, the distance (meters) from the treatment site is also indicated either for albumin (“alb”) or casein (“cas”). Arrows designate the zones in which treatments with casein (dashed dark red arrow) and albumin (blue arrow) were applied.

The percentages of marked *H. halys* in the eight zones of Area 4, for the period from 19 April to 17 May and from 18 May to 22 June are reported in Figure 8a,b, respectively. Milk solution was sprayed three times in the same overwintering site, whereas egg white solution was sprayed two times on plants considered as host of *H. halys* in each period (Table 2). From late April to mid-May, adults marked with casein or adults marked with albumin were found up to 450 m and 670 m away from the zone of treatment, respectively (Figure 8a). From late May to late June, the maximum distance at which marked adults were collected increased up to 510 m for casein and 690 m for albumin (Figure 8b). Moreover, the total number of sampled adults almost doubled in the second period (from 55 to 91), and percentages of adults marked with albumin increased from 11% to 39%. Overall, 8% of tested adults were positive for both casein and albumin in the two considered periods. None of the tested adults was positive to casein on 22 June, 44 days after the treatment was applied on 9 May.

## 4. Discussion

In this study, relationships between *H. halys* and host plants, and adult dispersal capacity in the spring were investigated in four areas of north-western Italy. At a landscape scale, the general features of these areas, like climate, altitude, presence of forest margins, natural corridors among the crops, buildings, water courses and main roads, were quite homogeneous. Consequently, the relative abundance of *H. halys* was similar among the areas. Wallner et al. [25] found *H. halys* displayed strong associations with urban factors, such as railroads, and other landscape/land use features that included deciduous forests and wetlands. In our study, analyzing data of captures by each trap in relation to the distances from the closest source of water, main roads and buildings, no differences were recorded between the numbers of bugs collected by traps in the different zones in each area. The zones in the four areas were also similar in terms of the presence of available host plants, even if in different amounts. As suggested by Kirkpatrick et al. [26], the influence of host plants must be considered when interpreting how *H. halys* responds to pheromone-baited traps and, perhaps, the similar behavioral response to pheromonal stimuli recorded in each zone could also be attributed to similarities in host plant composition.

In our study, the attraction of adults to lures, at least immediately after overwintering, was confirmed by both trap captures and plant sampling. Kirkpartick et al. [26] estimated the plume reach for pheromone-baited sticky traps to be approximately 3 m. Indeed, all the sampled host plant species hosted higher numbers of *H. halys* when they were located in proximity of a pheromone-baited trap (less than 5 m). This corroborates that the pheromone affects foraging behavior, potentially increasing the overall acceptability of the host plant resource or reducing *H. halys* dispersal from them [26,27]. Moreover, commercial pheromone lures seemed to be effective immediately following their placement in the field, from early/mid-April. The early attractiveness to overwintered adults was previously investigated in other studies with contrasting results [19,28,29]. Such attractiveness might have important implications in early season management of *H. halys*, by placing the lure on a favorite host plant. The effectiveness of pheromones for the initial attraction of dispersed adults could be coupled with the retention time given by the plant, in order to apply targeted management strategies. For example, attract-and-kill strategy requires the target pest to be attracted and retained in a spatially limited location by using a high dosage of pheromones in combination with a host plant, where it can be controlled to successfully preserve the main crop [27]. Our results suggest, for instance, a list of favorite host plants, such as common ash, peach, poplar, and walnut, to be used for such an early season management.

Naturally, *H. halys* population levels are affected by the local climatic conditions. In northern Italy, adults successfully exit from overwintering refuges when daily maximum temperatures exceed 14 °C, and females start ovipositing in mid-May and continue until mid-August [30]. Similarly, overwintered adults were captured in traps from mid-April, with an average temperature of 14.2 °C, and first nymphs were trapped from mid-June onwards. The abundance of overwintered adults, starting from their exit from refuges until reproduction, is influenced by temperatures, since captures peaked in late April, which corresponded to higher mean temperatures recorded in each area. Then, independently of weather condition, usually from July, populations grew due to the gradual increase of nymphs, and reached the highest levels from mid-July, when overwintered adults and first generation individuals overlapped.

*Halyomorpha halys* adults that disperse from overwintering sites begin to arrive on host plants in April through to May, and temporarily use transitional host plants, such as trees within the forest edge habitats, before following the availability of fruiting structures and colonize crops [6,31]. Similarly, starting from mid-April, adults were sampled on wild plants, such as hybrid plane, European spindletree, walnut, oak and European elder, which were common in forest edge and were not fruiting at that period. Consistently with studies in the native area [6], from May, *H. halys* was found also on plants bearing fruit, particularly on peach, mulberry and apple, while from June, it spread to many wild and crop plants, reaching hazelnut orchards; therefore, more adults were sampled, due to the increased availability of fruiting plants. In particular, some species, such as common ash, maple, hazelnut, cherry, walnut, black locust and bloody dogwood, are both favorite host plants and widespread in natural corridors in agroecosystems of north-western Italy, therefore, worthy to be considered important for dispersal of *H. halys*.

Before crop colonization, some wild hosts are suitable not only for nutrition but also for reproduction [6,15]. For *H. halys* oviposition, the presence of fruiting structures seems to contribute to plant selection more than aggregation pheromone lures [32]. In June, nymphs were found only on a few plants, such as peach, ivy, walnut, red mulberry, wheat, black locust, false indigo-bush, European spindletree, common dogwood, maple, and hazelnut. In the investigated areas, hazelnut is both a wild plant widespread in natural habitats, and a crop plant growing in commercial orchards. This species was either one of the first favorite transitional host plants early in the season, and was also a reproductive host, usually from June, when fruits become available and also susceptible to stink bug damage [4,33]. Movement from and to hazelnut plants, both wild and cultivated, occurred across all the sampling period. Obviously, this fact further complicates the pest management in hazelnut orchards, due to the continuous crop colonization by adults, in addition to the presence of all pest stages from mid-June to mid-August, when the local grown variety is usually harvested.

The qualitative analysis of each area shows that the presence of *H. halys* on a given plant species may change in relation to the floral composition in the zone. For example, in Area 2 *H. halys* adults were never found on walnut, whereas they were collected on maple and European spindletree nearby. Furthermore, on cherry, their abundance was variable in some zones, in relation to neighboring host plants. Moreover, although sunflower is reported as a potential trap crop for *H. halys* on pepper [21,34], it was never collected on this crop species during the growing season; however, in Area 3 sunflower was grown next to hazelnut orchards, which were probably more attractive. This finding leads to the importance of considering each agroecosystem for its peculiarities and floral composition; in fact, the existence of a large variety of plant species seems to be more relevant than the presence of a single possible attractive host plant species.

In addition to identifying the favorite host plants, the assessment of dispersal behavior and distribution of overwintered adults in the agroecosystem is crucial to implement sustainable pest management strategies. In fact, active dispersal by adults was observed season-long resulting in crop injury with strong edge effects in multiple commodities at a regional scale [14]; however, there is a gap between the time period in which *H. halys* disperses from overwintering sites and when it appears in agricultural crops, specifically tree bearing fruit. The immunomarking-capture was already tested and used as a useful and relevant technique to study *H. halys* moving within an orchard or between adjacent crops [12,21]. Here, this technique was applied to a wider landscape scale, in order to track movements from overwintering refuges to early host plants, and then to crops, particularly hazelnut. Despite the increased study area, overall a high number of collected bugs (44%) was positive for the markers, indicating that this technique can be usefully adopted to study *H. halys* dispersal on a wider scale. Indeed, there was evidence of long-range capture of marked adults up to 950 m (for albumin) and 650 m (for casein), i.e., the maximum range of tested distances. Since there were no distances at which no marked adults were found, future studies should include a wider range of distances. Indeed, the surface area of the study areas reached up to 50 ha. This could mean that *H. halys* adults need a wider area for their first dispersal flights in search of plants to feed on, confirming its strong dispersal capacity [10], especially immediately after the overwintering period [9,12]. 

Moreover, numbers of marked adults did not decrease with distance; overall they were found in all zones of each area, in different amounts but irrespective of the distance. First of all, the results suggest how *H. halys* adults are capable of both short- and long-range dispersal from overwintering sites and/or early host plants to next host and cultivated plants. Second, their movement patterns may not depend mainly on the distance from the sources but on the ecosystem features, and plant host distribution and availability. Therefore, *H. halys* is likely to perform short- or long-distance flights, implying a large movement within the agroecosystem [12]. Additionally, in accordance with previous studies [12,21], in each area there was a fraction of adults marked with both proteins confirming the high mobility within the landscape. In our case, double-marked individuals demonstrate that *H. halys* appear to move from anthropic overwintering sites to early host plants, or more unlikely vice versa. Although a contamination between individuals cannot be completely excluded, it is unlikely. In fact, in spring, after leaving overwintering refuges, adults tend to disperse rather than to aggregate in large groups as during summer. Moreover, most adults were collected on traps, probably before they could form aggregations.

Although stability of the two markers over time was not specifically tested, ELISA results showed more than double albumin positive specimens than casein positive ones, in compliance to what previously observed [12,23,24]. Jones et al. [22] demonstrated that over a 19-day period significantly lower percentages of marked pear psylla adults were casein positive. In addition to casein stability over time, a second cause could be attributed to *H. halys* behavior. It is well known the potential for *H. halys* to be marked either directly or to a lesser extent through direct contact with a previously marked surface [12]. Having treated possible anthropic overwintering sites with milk solution, the chances to reach directly overwintering adults were quite limited as it was unlikely that adults coming from different sites could have passed by human-made structures. On the other hand, egg solution was sprayed on possible natural overwintering sites and early host plants, which could be likely visited by higher numbers of *H. halys*, after leaving anthropic sites and seeking plants to feed on. Indeed, higher amounts of albumin positives were generally collected in the second period (from mid-May onwards), when overwintered adults left the refuges, and were present on early host plants. The early-season movement pattern of *H. halys* was particularly evident in Area 3. During April, all marked insects were casein positive or double protein positive: they came from anthropic overwintering sites, either directly or passing by early host plants. From May, casein and double protein positive adults almost disappeared (with few double-marked specimens): overwintered population left refuges and spread on plants, as evidenced by the percentages of albumin positives doubled throughout the Area 3. Here, casein was sprayed on a suitable overwintering site, where relatively high percentages of adults were reached (on average 27%), while albumin was sprayed on favorite early host plants (ivy, black locust, oak, chestnut) which were not natural overwintering sites. This result corroborates the potential of the immunomarking-capture technique to study dispersal behavior of *H. halys*, as assessed for other species [23].

## 5. Conclusions

This research provided information on early host plants and dispersal behavior of *H. halys* before crop colonization. Depending on ecosystem features and host plant availability, overwintered adults were capable of both short- and long-range dispersal, as assessed by immunomarking-capture. In addition to early host plants, *H. halys* adults proved to respond effectively to pheromone as soon as they left overwintering refuges. Therefore, the attractiveness of commercial lures coupled with the retention time given by the plants can be exploited to manage the pest early in the season.

## Figures and Tables

**Table 1:** Description of the four investigated areas in north-western Italy in 2018, and details on main features of zones enclosing pheromone-baited traps. For each area, the surface area of the adjacent forest together with its position (cardinal points) are indicated.

Area Characteristics				Sampling Period (No. of Samplings)	Trap Details				
#	Location	Surface Area (ha)	Forest (ha)		#	Surrounding Crops/Tree Rows/Forest	Min. Distance (m) from		
							Water	Road	Build.
1	Antignano (AT) N: 44°50′ E: 8°08′ 260 m a.s.l.	13.6	61.4 S-SE-SW	traps: 17 Apr–4 Jul (6) plants: 23 Apr–18 Jun (5)	1	forest	338	106	81
					2	hazelnut, forest	114	167	185
					3	hazelnut	218	37	50
					4	hazelnut, meadow, wheat	533	36	101
					5	vineyard, maize, apple	501	80	55
					6	maize, meadow	430	44	35
2	Frossasco (TO) N: 44°56′ E: 7°21′ 376 m a.s.l.	28.1	30.9 N; middle	traps: 5 Apr–4 Jul (7) plants: 20 Apr–22 Jun (6)	1	hazelnut, meadow	888	538	135
					2	wheat, meadow, forest	1002	604	277
					3	meadow, ryegrass, forest	841	1080	317
					4	meadow, ryegrass, forest	603	1140	243
					5	maize	486	967	113
					6	maize	446	753	195
3	Moncestino (AL) N: 45°9′ E: 8°09′ 287 m a.s.l.	48.1	147.4S-SW	traps: 5 Apr–29 Jun (7) plants: 18 Apr–14 Jun (6)	1	hazelnut, poplar, sunflower	317	111	235
					2	maize, wheat, poplar, sorghum	516	229	193
					3	maize	134	234	110
					4	maize	245	33	174
					5	forest	440	41	168
					6	maize, forest	507	202	315
4	Narzole (CN) N: 44°36′ E: 7°52′ 325 m a.s.l.	50.5	86.6 E-NE-N; SW	traps: 6 Apr–4 Jul (8) plants: 19 Apr–22 Jun (7)	1	hazelnut, alfalfa	1	108	209
					2	hazelnut, forest	118	277	284
					3	hazelnut, hybrid planes	15	844	161
					4	forest, hazelnut, maples	43	862	50
					5	hazelnut, forest, maples	5	838	235
					6	hazelnut, alfalfa, barley	1	436	301

**Table 2:** Details of the immunomarking-capture technique employed in the four areas.

Area	Date	Milk				Egg White				First Capture Date
		Site/Plant	Zone ^a^	Vol. (L) ^b^	Trap ^c^	Site/Plant	Zone ^a^	Vol. (L) ^b^	Trap ^c^	
1	17 Apr	rubble, woodshed	A	28	1	-				23 Apr
	23 Apr	-				hazelnut orchard	I	28	4	15 May
	15 May	peach trees	A	14	1	oak	H	28	6	23 May
2	20 Apr	hazelnut orchard	A	28	1	wild hazelnut	E	28	4	26 Apr
3	5 Apr	rubble,	H	14	-	-				18 Apr
		woodshed		14						
	18 Apr	rubble,	H	14	-	ivy, black locust, oak, chestnut	E	14	5	27 Apr
		woodshed		14			F	14	6	
4	6 Apr	shelter, woodshed	H	28	-	-				19 Apr
	19 Apr	shelter, woodshed	H	28	-	ivy, oak, ash	E	14	4	26 Apr
	9 May	shelter, woodshed	H	28	-	-				17 May
	17 May	-				cherry, apple, blackthorn	E	14	4	21 May

^a^: capital letters refer to zones identified in Figure S1. ^b^: volume of the sprayed solution. ^c^: the closest trap in the surroundings of a treated zone, if present.

**Table 3:** Mean numbers (±SE) of *Halyomorpha halys* adults per plant and per sampling period, collected on host plants in the four study areas from mid-April to mid-June. Abundance of *H. halys* referred to each 10-day period is given as qualitative index as follows: - = absence; + = 0.1–1; ++ = 1.1–4; +++ ≥ 4.1 mean adults/plant (sampling unit). Shading indicates the fruiting period of plants. Areas in which plants were sampled are also given; bold areas indicate the presence of bugs on the plant in that area.

Host Plants			April		May			June		Mean No.	Area of Presence
Common Name	Family	Scientific Name	II	III	I	II	III	I	II		
peach	Rosaceae	*Prunus persica* (L.) Batsch		-		++	-	+++	++	3.07 ± 2.06	**1**
mulberry	Moraceae	*Morus rubra* L.			-	-	++	++	++	1.63 ± 0.78	**1**, 2
apple	Rosaceae	*Malus domestica* Borkh.				+	-		++	1.33 ± 0.88	**1**, 2, 4
common ash	Oleaceae	*Fraxinus excelsior* L.	-	-	++	-	+	+++	+	1.24 ± 0.70	**2**, **4**
maple	Sapindaceae	*Acer* spp.	-	-	-	-	-	++	++	0.63 ± 0.40	1, **2**, 3, **4**
hybrid plane	Platanaceae	*Platanus hispanica* Mill.	++	-		-	-		-	0.60 ± 0.60	**4**
American pokeweed	Phytolaccaceae	*Phytolacca americana* L.						+	-	0.50 ± 0.50	**2**
sorrel	Polygonaceae	*Rumex* spp.			+	-				0.50 ± 0.50	**3**, **4**
vetch	Fabaceae	*Vicia* spp.			+	-				0.50 ± 0.50	**4**
hazelnut	Betulaceae	*Corylus avellana* L.	-	-	-	-	-	+	++	0.40 ± 0.32	**1**, **2**, **3**, **4**
cherry	Rosaceae	*Prunus avium* (L.) L.	-	-	+	+	+	+	+	0.34 ± 0.09	**1**, **2**, 3, **4**
bay	Lauraceae	*Laurus nobilis* L.		-		-		+		0.33 ± 0.33	**1**
black poplar	Salicaceae	*Populus* spp.	-	-	++	-	-	-		0.33 ± 0.33	**2, 3,** 4
black walnut	Juglandaceae	*Juglans nigra* L.				+	-		-	0.33 ± 0.33	**2**, **3**
wild hop	Cannabaceae	*Humulus lupulus* L.			-	+		-		0.33 ± 0.33	**1,** 2, 3
European spindletree	Celastraceae	*Euonymus europaeus* L.	-	+	+		+	-	+	0.33 ± 0.12	**2**, **3**
ivy	Araliaceae	*Hedera helix* L.	-	-	-	-	+	++	-	0.30 ± 0.20	**1**, 3, **4**
walnut	Juglandaceae	*Juglans regia* L.	-	+	+	+	+	+	-	0.29 ± 0.11	**1**, 2, **3**, 4
fig	Moraceae	*Ficus carica* L.		-		-	++	-	-	0.27 ± 0.27	**1**
false indigo-bush	Fabaceae	*Amorpha fruticosa* L.	-		-	+	-		-	0.20 ± 0.20	**3**
black locust	Fabaceae	*Robinia pseudoacacia* L.	-	-	+	+	+	-	+	0.18 ± 0.08	**1**, **2**, **3**, 4
common wheat	Poaceae	*Triticum aestivum* L.	-		+	-	-	-	+	0.17 ± 0.11	**2**, 3
European elder	Adoxaceae	*Sambucus nigra* L.	-	+	+	-	-	-	+	0.16 ± 0.10	1, **2**, 3, **4**
bloody dogwood	Cornaceae	*Cornus sanguinea* L.	-	-	+	-	+	+	-	0.14 ± 0.07	1, **2**, **3**, **4**
elm	Ulmaceae	*Ulmus minor* Mill.	-	-		-	-	+	+	0.13 ± 0.09	**1, 2**, 3, 4
oak	Fagaceae	*Quercus* spp.	-	+	+	+	-	-	-	0.11 ± 0.07	**1**, **2**, 3, 4
sand grape	Vitaceae	*Vitis rupestris* Scheele			-	-	-	+	-	0.10 ± 0.10	**1**, 2, 3
blackthorn	Rosaceae	*Prunus spinosa* L.		-	-	-	-	-	+	0.08 ± 0.08	1, **2**
willow	Salicaceae	*Salix* spp.	-	-	-	+	-	-	-	0.07 ± 0.07	2, **3,** 4

## Supplementary Materials

The following are available online at https://www.mdpi.com/2075-4450/11/12/866/s1, Figure S1: Aerial maps of the four investigated Areas.
