# Supplementary material for: First Insights on Early Host Plants and Dispersal Behavior of Halyomorpha halys (Hemiptera: Pentatomidae) from Overwintering to Crop Colonization
Source: Insects. 2020 Dec 6;11(12):866. doi: 10.3390/insects11120866 (PMC7762184; doi:10.3390/insects11120866)
Supplement: Supplementary file 1 [file insects-11-00866-s001.pdf]

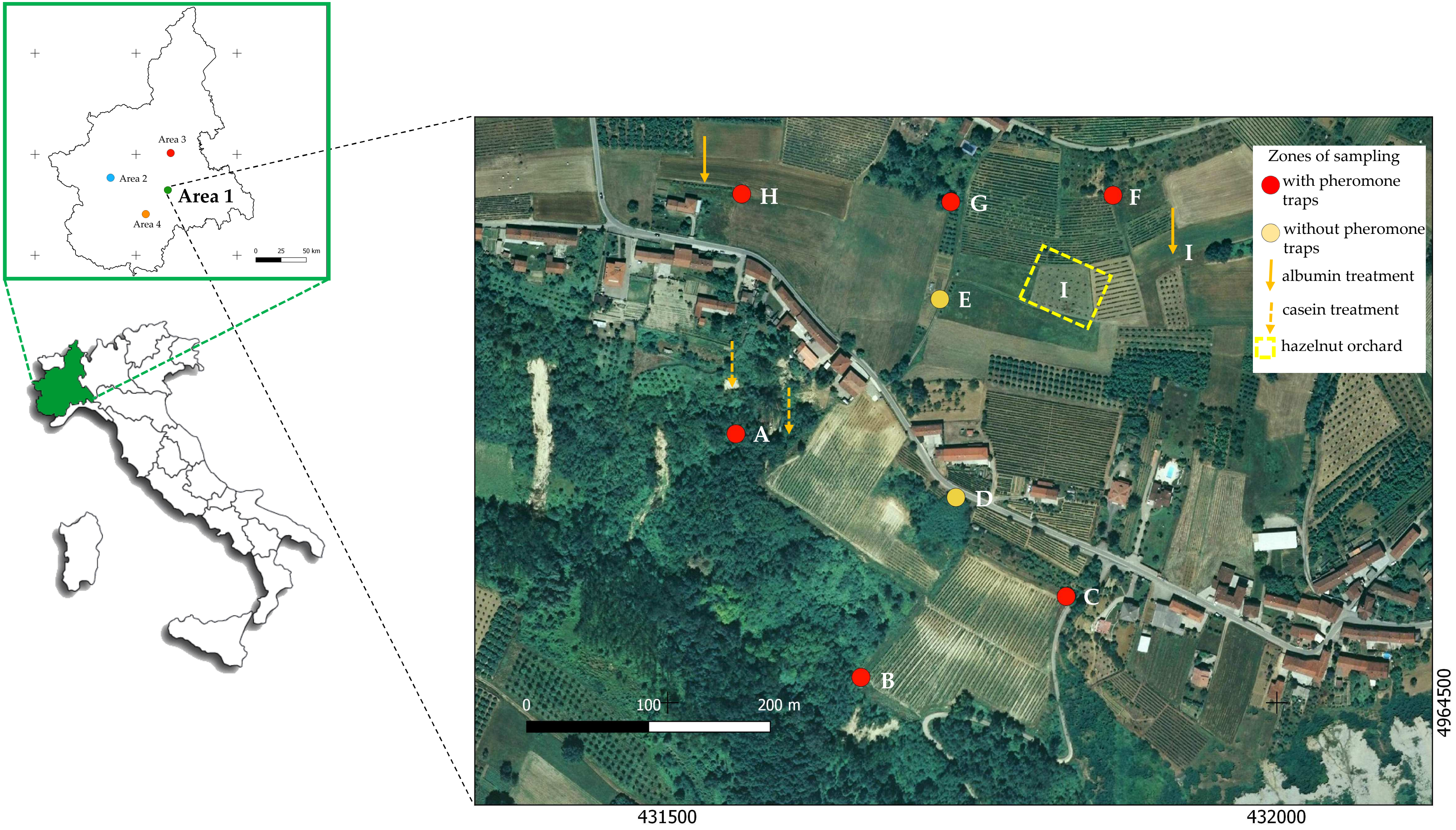
Area 3
Area 2
Area 1
Area 4
0
25
50 km
Zones of sampling
with pheromone traps
without pheromone traps
albumin treatment
casein treatment
hazelnut orchard
H
G
F
I
E
A
D
C
B
0
100
200 m
431500
432000
4964500

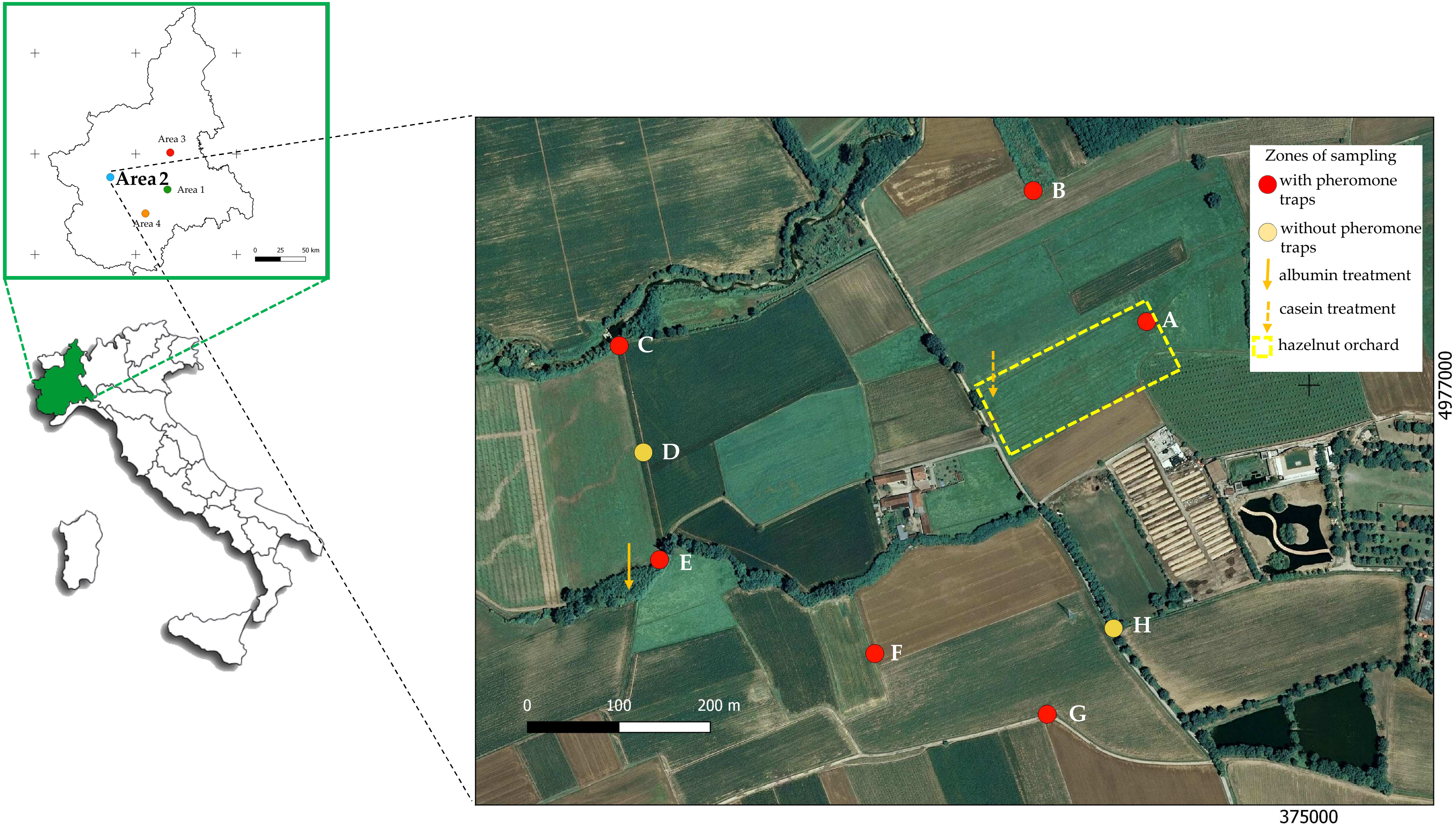

Area 3
Area 2
Area 1
Area 4
0
25
50 km
B
A
C
D
E
F
G
H
Zones of sampling
with pheromone traps
without pheromone traps
albumin treatment
casein treatment
hazelnut orchard
4977000
375000
0
100
200 m

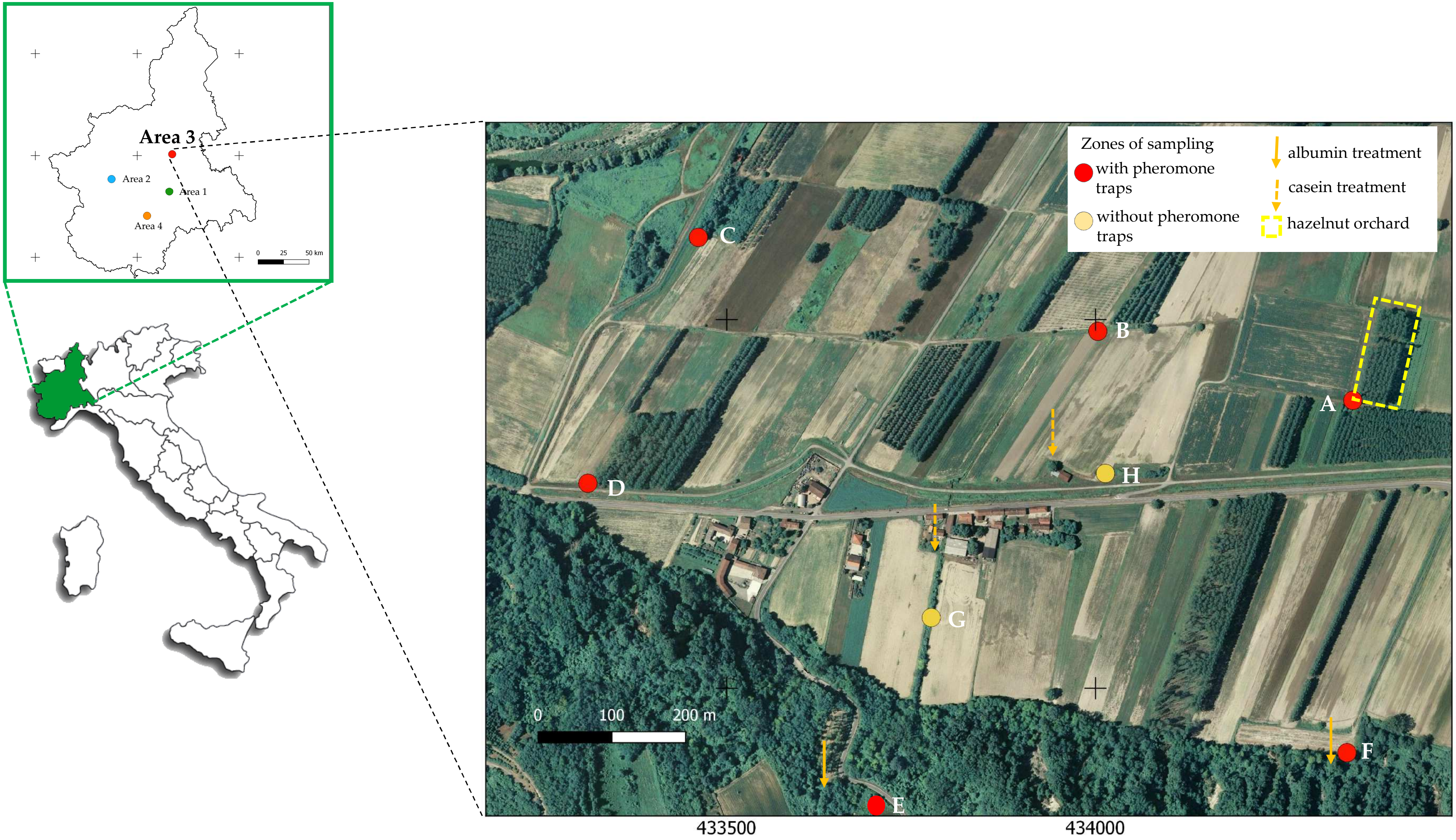
Area 3
Area 2
Area 1
Area 4
0 25 50 km
Zones of sampling
with pheromone traps
without pheromone traps
albumin treatment
casein treatment
hazelnut orchard
A
B
C
D
E
F
G
H
0 100 200 m
433500
434000
5001500
5001000

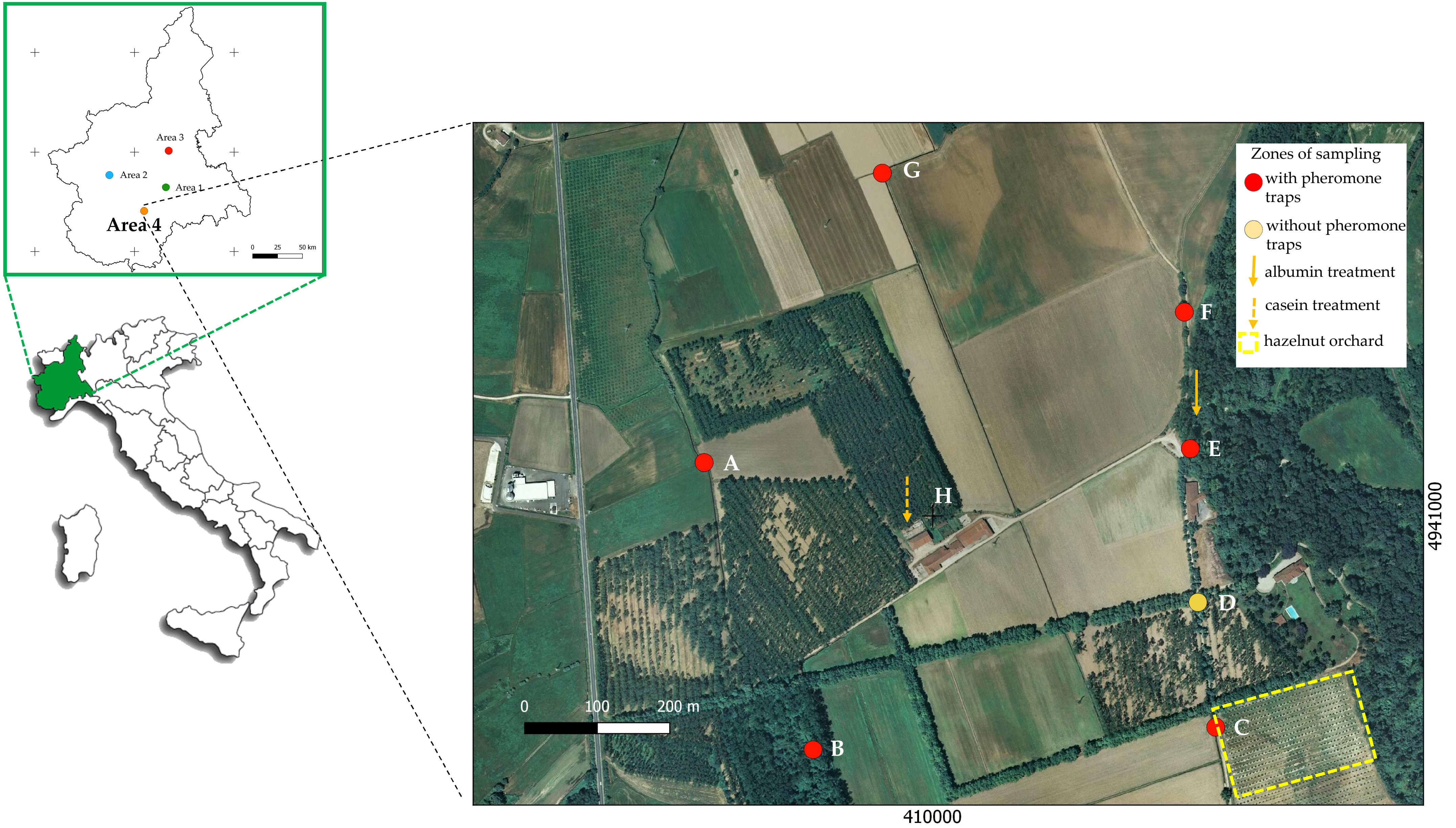

Area 3
Area 2
Area 1
Area 4
0 25 50 km
Zones of sampling
with pheromone traps
without pheromone traps
albumin treatment
casein treatment
hazelnut orchard
G
F
E
A
H
D
C
B
0 100 200 m
410000
4941000
